# Effects of the Dietary Fat Concentration and Fatty Acid Pattern on the Urine Composition, Apparent Nutrient Digestibility, and Selected Blood Values of Healthy Adult Cats

**DOI:** 10.3390/metabo14110605

**Published:** 2024-11-08

**Authors:** Nadine Paßlack, Simon Franz Müller, Kathrin Büttner, Jürgen Zentek

**Affiliations:** 1 Small Animal Clinic, Faculty of Veterinary Medicine, Justus-Liebig-University Giessen, 35392 Giessen, Germany; 2 Laboklin GmbH & Co. KG, 97688 Bad Kissingen, Germany; 3 Unit for Biomathematics and Data Processing, Justus-Liebig-University Giessen, 35392 Giessen, Germany; kathrin.buettner@vetmed.uni-giessen.de; 4 Institute of Animal Nutrition, Department of Veterinary Medicine, Freie Universität Berlin, 14195 Berlin, Germany; juergen.zentek@fu-berlin.de

**Keywords:** n-3 fatty acids, calcium oxalate, triglycerides, cholesterol, feline

## Abstract

**Background/Objectives**: The dietary fat concentration and fatty acid profile can influence various aspects of the feline organism. This study examined their effects on the urine composition, apparent nutrient digestibility, and selected blood variables. **Methods**: Ten healthy adult cats (46.6 ± 14.1 months old, initial body weight 4.99 ± 0.91 kg) received a low-fat basic diet with or without the addition of sunflower oil, fish oil, or lard in a randomized crossover design. The oil and lard were added to the daily amount of food at 0.5 or 1 g/kg body weight of the cats. At the end of each 3-week feeding period, urine, feces, and fasting blood samples were collected. **Results**: The results demonstrated only small effects of the dietary fat concentration and source on the urine composition of the cats. In addition, the apparent nutrient digestibility was unaffected by the dietary treatments. The supplementation with fish oil, but not sunflower oil or lard, lowered the triglycerides and increased the total and low-density lipoprotein cholesterol concentrations in the plasma of the cats (*p* < 0.05). However, these blood values were within the physiological reference ranges among all groups. **Conclusions**: It can be concluded that the dietary fat content and fatty acid profile did not adversely affect the urine composition or nutrient digestibility in healthy adult cats. The lipid metabolism of the animals was modulated by the supplementation with fish oil, a relevant source of n-3 fatty acids. The observed triglyceride-lowering effect should be further investigated in clinical studies.

## 1. Introduction

In human medicine, the dietary fat concentration is discussed as one factor in the pathogenesis of calcium oxalate stones, as high fat intakes have been associated with increased urinary oxalate excretion [1,2,3]. The potential underlying mechanisms include intestinal binding between calcium and fatty acids, which could reduce the formation of calcium oxalate complexes in the gut and, thus, increase the intestinal absorption of oxalate [4]. This metabolic disorder is referred to as “enteric hyperoxaluria” [5]. Moreover, it is assumed that high fat intakes could enhance the hepatic activity of lactate dehydrogenase and other enzymes, resulting in increased endogenous oxalate synthesis [3]. In addition to the impact of the overall fat intake, a role of specific fatty acids, particularly arachidonic acid (20:4 n-6), is also discussed in the pathogenesis of calcium oxalate stones. It has been demonstrated that arachidonic acid dose-dependently increases the phosphorylation of the erythrocyte band 3 protein [6], a transport protein, which catalyzes the exchange of anions, such as oxalate, across the red blood cell membrane [7]. As one consequence, increased phosphorylation of the erythrocyte band 3 protein in the intestine and kidneys may enhance the intestinal oxalate absorption and renal oxalate excretion [8,9].

Calcium oxalate urinary stones are also a significant health issue in cats [10,11,12], and dietary strategies to prevent this type of urolith are currently being researched. However, there are still limited data on the impact of dietary fat on the formation of calcium oxalate stones in felines. In a retrospective study by Dijcker et al. [13], the impact of 252 dry diets on the urinary oxalate concentration and excretion was assessed in a cohort of 65 cats. The dietary crude fat concentrations ranged between 1.66 and 5.58 g/100 kcal (corresponding to 0.40–1.33 g/100 kJ, or to 5.95–20.0 g/100 g dry diet, with an energy density of 1.5 MJ ME/100 g). A multivariate linear regression analysis demonstrated a weak and negative association of the dietary crude fat concentration with the urinary oxalate excretion in cats [13]. On the other hand, a case–control study by Lekcharoensuk et al. [14], which was based on an evaluation of the dietary history of 173 cats with calcium oxalate uroliths, 290 cats with struvite uroliths, and 827 control cats, revealed that a moderate fat concentration in the diet (5.03–5.24 g/100 kcal, corresponding to 1.20–1.25 g/100 kJ, or to 18.0–18.8 g/100 g dry diet, with an energy density of 1.5 MJ ME/100 g) was associated with an increased risk of calcium oxalate stones [14]. Zentek and Schulz [15] detected an increase in renal oxalate excretion in cats fed low-protein/high-fat diets (21.7–27.3% crude protein and 21.8–23.5% crude fat in dry matter) compared to high-protein/low-fat diets (64.0–77.6% crude protein and 10.1–16.9% crude fat in dry matter). In a study by Dijcker et al. [16], three diets with either a high carbohydrate (34.75 g nitrogen-free extracts/MJ ME), high protein (45.43 g crude protein/MJ ME), or high fat concentration (18.95 g lipids/MJ ME) were evaluated in cats. The authors found that a high carbohydrate or a high fat content in the diet led to higher urinary oxalate concentrations than a high dietary protein content [16]. Overall, the present, albeit limited, data may indicate a tendency toward a higher risk of calcium oxalate stones in cats with greater fat intakes. However, dose-dependency studies on the effects of varying dietary fat concentrations on the feline urine composition are lacking. Moreover, it has not yet been investigated whether the dietary fatty acid pattern may also play a role in the pathogenesis of calcium oxalate stones in cats.

It was therefore the aim of the present study to evaluate the relevance of the dietary fat concentration and fatty acid pattern for the renal excretion of oxalate in cats in more detail. For this, different amounts of selected fat sources were supplemented in a feeding trial: sunflower oil (rich in n-6 polyunsaturated fatty acids), fish oil (rich in n-3 polyunsaturated fatty acids, but also a source of arachidonic acid), and lard (rich in saturated and monounsaturated fatty acids, and also a source of arachidonic acid). Based on the available studies, it was hypothesized that the overall fat intake, but also the dietary n-6:n-3 ratio or the provision of specific fatty acids (particularly arachidonic acid), could modulate renal oxalate excretion in felines. Moreover, in order to assess possible side effects of higher fat intakes or varying dietary fatty acid profiles, the blood values of the cats were checked routinely, additional urinary parameters were measured, and the apparent nutrient digestibility was determined.

## 2. Materials and Methods

This study was approved by the relevant local authority in Giessen, Germany (“Regierungspräsidium Giessen”, ethical approval code G41/2022).

### 2.1. Animals and Diets

Ten healthy adult colony cats (3 male neutered and 7 female neutered) from the Small Animal Clinic of the Justus-Liebig-University Giessen, aged 46.6 ± 14.1 months and with an initial body weight of 4.99 ± 0.91 kg, were included in the investigation, using a randomized crossover design. The group size was based on a power calculation using G*Power 3 [17].

In total, 7 dietary treatments were considered, where each feeding period lasted for 21 days. The animals received a commercial complete low-fat diet (Vet-Concept, Cat Intestinal Low Fat, Föhren, Germany; analyzed crude fat concentration: 9.07 g/100 g dry matter) without (w/o; control treatment) or with the addition of sunflower oil (Aro, MCC Trading International GmbH), fish oil (Grizzly Lachsöl, Grizzly Pet Products, Woodinville, WA, USA), or lard (purchased from a private butcher in Ulrichstein Feldkrücken, Germany). The oils and lard were added to the daily amount of food at a concentration of 0.5 g or 1.0 g per kg body weight (BW) of the cats. In this way, three graduated dietary fat levels could be evaluated, achieving approximately 9%, 12%, and 15% crude fat in dry matter for the control treatment and after the addition of 0.5 and 1.0 g oil/lard per kg BW of the cats, respectively. For palatability reasons, no higher supplementation doses could be applied.

Due to the randomized crossover design, the order of the dietary treatments differed among the individual cats during the study period. The setup was in accordance with the general principles of a Latin square design (7 × 7), but with 3 pairs of cats receiving the same treatment owing to the study group of n = 10. In addition, the same oil/fat was not supplemented in the consecutive feeding period, so as to evaluate dose-dependent effects of the supplementation without a possible interference of time (Table 1).

The diet, oils, and lard were purchased from one batch only. Deionized water was offered ad libitum throughout the study.

The amounts of food were calculated to cover the energy requirements of cats [18] and individually adjusted on a weekly basis in order to maintain the BW of the animals. The feed intake of the cats was recorded daily, and their BW was recorded weekly. During the collection periods, the individual water intake of the animals was also assessed.

The dry matter and nutrient concentrations in the basic diet, as well as the fatty acid composition of the basic diet, oils, and lard, were analyzed by an accredited laboratory (AGROLAB LUFA GmbH, Kiel, Germany) using the official methods for feed analysis (moisture and crude nutrients: Commission Regulation (EC) No 152/2009 III, A, M, C, I: 2009-01 and Commission Regulation (EC) No 152/2009 III, H, Procedure B: 2009-01; minerals: DIN EN 15,621: 2017-10; fatty acids: German Society for Fat Science (DGF) C-VI 11a: 2016 (mod.) + DGF C-VI 10a: 2016 (mod.)). For the fatty acid analyses, a gas chromatography system (7890 A or a comparable model available in the commercial laboratory, Agilent Technologies, Waldbronn, Germany) was used. The column used to separate the fatty acids was a CP Sil 88 (50 m length, 0.25 mm inner diameter; 0.2 µm film thickness, Agilent Technologies, Waldbronn, Germany). Hydrogen was used as a gas carrier, and as a certified standard to control the response of the gas chromatograph. A standard mixture was additionally applied in each measuring sequence to control the separation of the fatty acids.

The dietary oxalate concentration was determined as described previously [19], using a commercial test kit (oxalate oxidase assay EnzytecTM Oxalsäure, no. E2100; R-Biopharm AG, Darmstadt, Germany). For the sample preparation, 1 g of the diet was first mixed with 4 mL of hydrochloric acid (5 M) (15 min; Multi Reax, Heidolph Instruments GmbH & Co., Schwabach, Germany). The solution was then heated (3 h, 60 °C; Haake C10-W13, Thermo Scientific, Karlsruhe, Germany) and centrifuged afterwards (15 min, room temperature, 2663× *g*; Heraeus Labofuge 400 R, Thermo Scientific, Karlsruhe, Germany). The supernatant was filtered (SCFA Syringe Filter, 0.2 μm; Thermo Scientific, Karlsruhe, Germany), and a 500 μL aliquot of the filtered solution was consecutively adjusted to a pH of 2.9–3.1 with either sodium hydroxide or hydrochloric acid. After another centrifugation (10 min, room temperature, 17,000× *g*; Heraeus Fresco 17 Centrifuge; Thermo Scientific, Karlsruhe, Germany), 10 μL of the supernatant was mixed with 200 μL of Reagent 1 included in the commercial test kit (1 min, 37 °C, 1050 rpm, BioShake iQ, Analytic Jena, Jena, Germany). The solution was then incubated (5 min, 37 °C, 590 nm; TECAN infinite M200 PRO; Tecan Group Ltd., Crailsheim, Germany), followed by the addition of 20 μL of Reagent 2 from the test kit. After mixture (1 min, 37 °C, 1050 rpm; BioShake iQ; Analytic Jena, Jena, Germany) and incubation (15 min, 37 °C, 590 nm; TECAN infinite M200 PRO; Tecan Group Ltd., Crailsheim, Germany), the extinction could be measured (TECAN infinite M200 PRO; Tecan Group Ltd., Crailsheim, Germany). The resulting oxalate concentration in the sample was calculated as specified by the manufacturer of the test kit.

The measured dietary dry matter and nutrient concentrations are presented in Table 2, and the fatty acid patterns of the basic diet, oils, and lard are provided in Table 3.

### 2.2. Housing and Sample Collection

**Figure 1:**
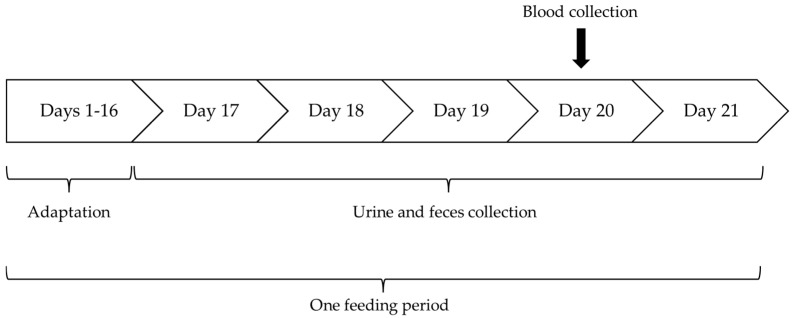
Time schedule of a feeding period: In total, seven feeding periods of 21 days each were considered in the present study (Table 1 and description in Section 2.1 and Section 2.2). Each feeding period was divided into an adaptation period (days 1–16) and a sample collection period (days 17–21).

The cats were housed in one group throughout the study, with the exception of the urine and feces collection periods. After adaptation to the respective dietary treatment for 16 days, the cats were individually housed in comfortable metabolic cages (length: 1.7 m, width: 0.9 m, height: 1.25 m; additional second floor of 0.6 m^2^) for the next 5 days to collect their urine and feces (Figure 1). For this, the cat litter boxes were prepared with plastic pellets as litter. The samples were removed several times a day and stored at −20 °C (urine) or −80 °C (feces) until further analyses. On the penultimate day of each sampling period, fasting (≥14 h) blood was collected in the morning by puncture of the *Vena cephalica antebrachii*, using sterile disposable needles (Sterican^®^, Braun, Melsungen, Germany) and lithium heparin-coated tubes (S-Monovette^®^ Lithium Heparin, Sarstedt, Nümbrecht, Germany). The blood was centrifuged (1900× *g*, 4 °C, 10 min; Heraeus Megafuge 16 R, Thermo-Fisher Scientific, Karlsruhe, Germany), and the plasma obtained was stored at −80 °C. The samples were sent frozen to an external laboratory (see Section 2.4 *Blood analyses*) and kept frozen until the day of analysis.

### 2.3. Urine and Feces Analyses

The urine and feces samples of the cats were analyzed as described previously [20]. In short, the urinary pH was measured with a pH meter (SevenMulti pH meter, Mettler-Toledo GmbH, Schwerzenbach, Switzerland), and the urinary anions and cations were measured using ion-exchange high-performance liquid chromatography systems (Dionex DX-500 and Dionex DX-120; Dionex Corporation, Sunnyvale, CA, USA). The urea and creatinine concentrations in the urine were determined by high-performance liquid chromatography (Agilent 1100 with an UV detector, Agilent Technologies, Waldbronn, Germany). The relative supersaturation of the urine was calculated using the Supersat program [21].

In the fecal samples, the chloride concentrations were analyzed by ion-exchange high-performance liquid chromatography (Dionex DX-500; Dionex Corporation, Sunnyvale, CA, USA), and the concentrations of phosphorus were measured with a spectrophotometer (Ultrospec 2000, Pharmacia Biotech, Cambridge, UK). The remaining mineral concentrations were measured with an atomic absorption spectrometer (type contrAA 800, Analytik Jena AG, Jena, Germany).

The apparent nutrient digestibility (%) was calculated as follows: ((mineral intake (mg/d)-fecal mineral excretion (mg/d))/mineral intake (mg/d)) * 100.

The fecal score of the cats was assessed daily during the collection periods, using a 1–5-point scale (1 = very hard/dry; 5 = liquid; [22]), where a score of 2–3 was considered to represent physiological fecal consistency.

### 2.4. Blood Analyses

The plasma samples of the cats were analyzed by an accredited commercial veterinary diagnostics laboratory (Laboklin, Bad Kissingen, Germany), using accredited standard methods.

The alkaline phosphatase and lactate dehydrogenase activities, as well as the concentrations of cholesterol, high-density lipoprotein cholesterol, low-density lipoprotein cholesterol, triglycerides, non-esterified fatty acids, total protein, urea, creatinine, calcium, and phosphate, were measured with a COBAS 8000 modular analyzer system (Roche, Mannheim, Germany) by a cobas c-type module. For each respective parameter, a corresponding commercial test kit (Roche, Mannheim, Germany) specifically designed for the analyte was used. The respective concentrations of the analytes were determined by photometric measurements within the cobas c module. All kits underwent daily calibration controls in the laboratory according to accreditation and quality assurance guidelines and were validated for the measurement of cat samples.

On the day of analysis, all samples were measured as one batch and for each parameter with a single kit to exclude any day-to-day or lot-to-lot variations.

### 2.5. Statistics

For the statistical data analyses, the programs SPSS 28 (IBM Corp; Released 2021; IBM SPSS Statistics for Windows, Armonk, NY, USA) and SAS 9.4 (SAS^®^ Institute Inc., 2013; Base SAS^®^ 9.4 Procedures Guide: Statistical Procedures, 2nd edition ed. Statistical Analysis System Institute Inc., Cary, NC, USA) were used for the descriptive analyses and group comparisons, respectively. A repeated-measures ANOVA was carried out separately for each treatment (sunflower oil, fish oil, lard), where the basic diet without supplementation was compared with the supplementation of 0.5 g and 1 g of oil/lard per kg BW/day (3 doses each, 10 cats per group). The pairwise comparisons were adjusted with the Bonferroni correction. The data are presented as means and pooled standard error of the means. The statistical significance was set at *p* < 0.05.

## 3. Results

### 3.1. Health, Feed and Water Intake, and BW

The cats were healthy throughout the study, with the exception of one cat with a respiratory disorder during two feeding periods (“w/o” and “0.5 g lard/kg BW/day”). This cat was removed from the ongoing feeding trial until recovery.

In addition, as lard from a single batch had to be used for the study to ensure a constant fatty acid pattern, two cats were excluded from the 1 g lard/kg BW/day treatment during the study, as the available amount of lard was limited.

The feed and water intake, as well as the BW of the cats, did not differ among the treatment groups (*p* > 0.05; Table 4).

### 3.2. Urine Composition and Renal Excretion

The dietary fat content and fatty acid pattern only slightly affected the urine composition of the cats and did not change their renal mineral excretion (Table 5 and Table 6). The urinary citrate concentrations were higher when the cats received 1 g lard/kg BW/day than 0.5 g lard/kg BW/day (*p* = 0.0008 for the overall lard treatment and *p* = 0.0441 for the pairwise comparison). In addition, the concentrations of hippuric acid in the urine were lower when the diet was supplemented with 1 g sunflower oil/kg BW/day than with 0.5 g sunflower oil/kg BW/day (*p* = 0.0403 for the overall sunflower oil treatment and *p* = 0.0408 for the pairwise comparison).

The calculated relative supersaturation of the urine with magnesium ammonium phosphate (struvite) and calcium oxalate did not differ between dietary treatments (*p* > 0.05), remaining within the range of a metastable zone (calcium oxalate) or oversaturation (struvite) [23].

### 3.3. Feces Composition, Fecal Excretion, and Apparent Nutrient Digestibility

The fecal scores and dry matter concentrations were not different among the groups (*p* > 0.05; Table 7). With increasing dietary fat levels, the fat concentrations in the feces of the cats also increased (*p* = 0.0044, *p* = 0.0029, and *p* = 0.0358 for the overall sunflower oil, fish oil, and lard treatments, respectively; *p* = 0.0046 for the pairwise comparison “w/o” versus “1.0 g sunflower oil/kg BW/day” and *p* = 0.0034 for the pairwise comparison “w/o” versus “1.0 g fish oil/kg BW/day”). The phosphorus concentrations were decreased in the feces at higher fat intakes of the animals (*p* = 0.0407, *p* = 0.0213, and *p* = 0.0120 for the overall sunflower oil, fish oil, and lard treatments, respectively; *p* = 0.0264 for the pairwise comparison “w/o” versus “0.5 g fish oil/kg BW/day” and *p* = 0.0220 for the pairwise comparison “w/o” versus “1.0 g lard/kg BW/day”). Although statistically significant, however, these intergroup differences were only small. Small variations in the fecal crude ash (*p* = 0.0050 for the overall lard treatment and *p* = 0.0134 for the pairwise comparison “w/o” versus “1.0 g lard/kg BW/day”) and potassium concentrations (*p* = 0.0340 for the overall lard treatment) were also observed when lard was added to the basic diet, as well as a slightly higher calcium concentration in the feces when sunflower oil was included in the diet (*p* = 0.0423 for the overall sunflower oil treatment and *p* = 0.0435 for the pairwise comparison “w/o” versus “0.5 g sunflower oil/kg BW/day”).

The fecal nutrient excretion and the apparent nutrient digestibility were not affected by the dietary supplementation with sunflower oil, fish oil, or lard (*p* > 0.05; Table 8).

### 3.4. Clinical–Chemical Parameters in the Plasma

The measured clinical–chemical parameters in the plasma of the cats are presented in Table 9. With the exception of lactate dehydrogenase activity, all values were within the physiological reference range. However, the plasma activity of lactate dehydrogenase was only slightly above the reference range in the control group receiving the basic diet without additional fat supplementation, as well as in the treatment groups receiving the fish oil supplementation.

The addition of sunflower oil to the basic diet had no impact on the evaluated clinical–chemical parameters (*p* > 0.05), and the dietary supplementation with lard also revealed only small effects (*p* = 0.0008 and *p* = 0.0375 for the plasma concentrations of non-esterified fatty acids and creatinine, respectively). In contrast, the intake of fish oil resulted in several changes. The plasma total protein concentrations were lower when the cats received 1 g fish oil/kg BW/day instead of 0.5 g fish oil/kg BW per day (*p* = 0.0204), and the phosphate concentrations were higher when 1 g fish oil/kg BW/day was added to the diet compared to the control treatment (*p* = 0.0339). The lipid metabolism of the animals was also affected by the fish oil supplementation, as indicated by lower plasma triglyceride concentrations (0.5 g fish oil/kg BW/day versus control; *p* = 0.0305), higher total cholesterol concentrations (1 g fish oil/kg BW/day versus control; *p* = 0.0359), and higher low-density lipoprotein cholesterol concentrations (1 g fish oil/kg BW/day versus 0.5 g fish oil/kg BW/day; *p* = 0.0403) in the plasma.

## 4. Discussion

In the present study, the effects of varying fat concentrations and sources in a diet were evaluated in healthy adult cats. A low-fat diet was used as a basic treatment to assess the subsequent impact of the addition of sunflower oil (rich in n-6 polyunsaturated fatty acids), fish oil (rich in n-3 polyunsaturated fatty acids), or lard (rich in saturated and monounsaturated fatty acids) at different doses. The analyzed ratio of n-6:n-3 fatty acids in the basic diet was 6:1, which can be considered favorable for diets for cats [24]. The addition of the oils and lard to the basic diet resulted in a markedly different dietary fatty acid pattern. Moreover, varying dietary fat concentrations were assessed. In addition to a relatively low fat content in the basic diet (9% in dry matter), moderate-to-higher fat concentrations were evaluated by the addition of 0.5 g or 1.0 g oil/lard per kg BW of the cats. On condition that the daily amount of the basic diet had to be slightly reduced in these cases to ensure a comparable energy intake in all groups, approximate dietary fat concentrations of 12% and 15% in dry matter were achieved by the two supplementation doses.

Although previous studies in cats have indicated that a higher fat intake might be a risk factor for calcium oxalate uroliths [14,15,16], the results of the present investigation could not confirm this assumption. The urinary concentration and renal excretion of calcium and oxalate, as well as the relative supersaturation of the urine with calcium oxalate, were unaffected by the dietary fat content. Based on knowledge from human medicine, it could be supposed that high fat concentrations might interact with calcium in the intestine, preventing its complexation with oxalate and thereby enhancing the intestinal oxalate absorption [4]. However, the results on the urine composition of the cats, as well as on their apparent calcium digestibility and their calcium and oxalate excretion, do not support this mechanism in felines. It should be considered that the oxalate concentration in cat food might be relatively low. High amounts of oxalate are especially found in green vegetables, while meat and fish contain only low amounts [25]. Thus, a typical carnivorous diet should not be rich in oxalate, as also applicable to the basic diet used in the present study. The low oxalate concentrations in cat food may therefore limit the intestinal complexation with calcium or the effects of interfering dietary factors, such as fat.

It has also been assumed that high dietary fat concentrations can affect the activity of lactate dehydrogenase or other enzymes involved in endogenous oxalate synthesis [3]. In the present study, the plasma activity of lactate dehydrogenase did not differ depending on the fat intake of the cats, which might be another explanation for the unaffected urinary oxalate concentrations. These results are also in line with our previous investigation, where we could not detect a difference in renal oxalate excretion between healthy control cats and cats with hyperlipidemia [26]. Thus, the fat metabolism of cats seems not to be closely linked with their oxalate metabolism.

Finally, arachidonic acid has been demonstrated to modulate the phosphorylation of the erythrocyte band 3 protein [6] and, therefore, the oxalate flux, which could contribute to higher urinary oxalate concentrations in humans [8]. In the present study, the erythrocyte band 3 protein was not specifically investigated. However, as the supplementation with fish oil and lard increased the amounts of arachidonic acid in the cats’ diet without changing their urinary oxalate concentrations, an effect of arachidonic acid on the oxalate metabolism of cats could not be proven by our investigation.

Overall, the present data indicate that neither the dietary fat concentration nor the fatty acid profile of the diet might be a specific risk factor for the development of calcium oxalate stones in cats. As a limitation of this study, however, it should be noted that the addition of oil or lard to the basic diet was accompanied by a slight reduction in the daily amounts of feed to ensure a similar energy intake in all dietary treatment groups. In addition, we could observe a slightly (but only numerically) lower feed acceptance in the cats fed the fat-supplemented diets. Although the statistical analyses could not detect any differences in the daily feed intake among the groups, the numerically lower feed—and, therefore, nutrient—intakes should be considered for careful data interpretation. Due to the small and only numerical differences in the feed intake, however, we do not assume a significant impact on the present results.

We also aimed to evaluate potential side effects that could be associated with higher fat intakes or specific fatty acid profiles in the diet. While the nutrient digestibility was not affected, some interesting results of the fish oil supplementation on the lipid metabolism of the cats were observed. Although not strictly dose-dependently, fish oil increased the total and low-density lipoprotein cholesterol concentrations and lowered the triglyceride concentrations in the plasma of the animals. These effects were not observed when sunflower oil or lard was added to the basic diet.

Only a few studies are available on the effects of the dietary fatty acid pattern on blood lipids in cats so far. In the most recent investigation by Jewell and Jackson [27], the dietary supplementation with fish oil (as a source of eicosapentaenoic acid and docosahexaenoic acid) and chicken liver (as a source of arachidonic acid) did not affect the blood triglyceride concentrations in healthy adult cats. The blood cholesterol levels were lower in the fish oil group than in the chicken liver group on days 56 and 84, but they were no different to the unsupplemented control animals. When the cholesterol levels on day 84 were compared with the initial blood values, a lowering effect could only be observed in the fish oil group, which was significantly different to the other two treatments [27]. In another study, the same authors observed a decrease in the blood triglyceride and cholesterol concentrations in cats receiving fish oil when compared to their baseline values [28]. Conversely, Angell et al. [29] could not detect any effect of sunflower oil, fish oil, or safflower oil in the diet on the blood cholesterol levels of cats. The triglyceride levels, however, were lower after 2 and 4 weeks in the fish oil group when compared to the baseline values, and they were also lower in weeks 0, 2, and 4 compared to the sunflower oil group and in weeks 2 and 4 compared to the safflower oil group [29]. Finally, Plantinga and Beynen [30] did not observe any effect of dietary sunflower or fish oil supplementation on the plasma triglyceride or cholesterol concentrations in cats.

In the studies described above, moderate to relatively high dietary fat concentrations were evaluated (16–22% in dry matter), where the fat levels were comparable among the respective treatment groups [27,28,29,30]. In contrast, the present study investigated the dose-dependent effects of fish oil supplementation, achieving dietary fat concentrations between 9 and 15% on a dry matter basis. These differences in the fat intake of the cats might partially explain the observed divergent results of the blood cholesterol and triglyceride concentrations compared to some previous investigations. In addition, it should be noted that fish oil can vary widely in its fatty acid composition [31] and might, therefore, act differently in the feline organism.

In human medicine, reports on the effects of n-3-fatty acid supplementation on blood cholesterol levels are also conflicting [32,33]. Therefore, further studies are required to specifically investigate potential mechanisms of modulation of the cholesterol pathways by n-3-fatty acids and potential interfering factors.

Lowering effects of fish oil supplementation on blood triglycerides in cats have been partially described in the available studies [28,29] and are also supported by the present results. This effect is in line with a huge number of studies on human subjects, as recently reviewed, among others, by Liu [34], Wang et al. [32], and Feingold [33]. The described underlying mechanisms of the triglyceride-lowering effects of n-3-fatty acids in humans include inhibition of fatty acid synthesis and stimulation of fatty acid oxidation in the liver by a complex modulation of transcription factors, enzymes, and receptors [33,34].

As a limitation of this study, no wash-out period was considered between the dietary treatments. A crossover design was used to reduce the individual variation in treatment response [35], and a Latin square approach was used to control extraneous variables [36]. Moreover, the same oil or fat was not added in the consecutive feeding period, in order to rule out any impact on the evaluated dose-dependent effects. However, potential carry-over effects cannot be prevented with this study design [35]. Nevertheless, data in human subjects have demonstrated that an impact of oral supplementation with n-3 fatty acid ethyl esters on plasma triacylglycerols and free fatty acids was already observed after 2–7 days but disappeared within one week after the end of the supplementation [37]. Thus, although no wash-out periods were included in the present study design, it can be assumed that the feeding periods were long enough to compensate for potential effects of the preceding feeding period, and to detect the specific metabolic effects of the current oil or fat supplementation.

Finally, for the interpretation of the present results, it should be noted that no long-term effects of varying fat concentrations and fatty acid patterns in a diet could be assessed with the underlying study design. A supplementation period of 3 weeks was adequate to detect the described effects on the lipid metabolism of cats. Previous investigations considered 4-week [28,29,30] or 12-week treatment periods [27] in this context. For future studies, however, it would also be interesting to evaluate possible longer-term adaptations of the feline organism to different fat intakes, including a greater variability in dietary fat concentrations than investigated in the present study.

Overall, clinical studies on supplementation with fish oil in cats with disturbances of the fat metabolism seem to be advisable to evaluate potential dietetic strategies in these patients. In this context, a preliminary study on the use of n-3-fatty acids in hyperlipidemic Schnauzer dogs has recently been published [38]. The authors found that the addition of fish oil to a low- or moderate-fat diet reduced the blood triglyceride and cholesterol levels in these patients. Whether such effects might also apply in cats with lipid disorders should be evaluated in future research.

## 5. Conclusions

The dietary fat concentration and fatty acid pattern seem not to be specific risk factors for the development of calcium oxalate uroliths in cats. The addition of fish oil as a source of n-3 fatty acids revealed lowering effects on blood triglycerides. It is recommendable to investigate the clinical relevance of this finding in future studies, taking a longer supplementation period and a greater variability in dietary fat concentrations into account.

## Figures and Tables

**Table 1:** Design of the present study: 10 cats were included in the investigation, receiving a low-fat basic diet without (“w/o”, control treatment) or with the addition of sunflower oil, fish oil, or lard. The oils and lard were added to the basic diet at concentrations of 0.5 g/kg BW/day (“0.5 g”) and 1 g/kg BW/day (“1.0 g”).

Cat (Number)	w/o	Sunflower Oil 0.5 g	Fish Oil 1.0 g	Lard 1.0 g	Fish Oil 0.5 g	Sunflower Oil 1.0 g	Lard 0.5 g
1	Feeding period 1	Feeding period 2	Feeding period 3	Feeding period 4	Feeding period 5	Feeding period 6	Feeding period 7
2	Feeding period 7	Feeding period 1	Feeding period 2	Feeding period 3	Feeding period 4	Feeding period 5	Feeding period 6
3	Feeding period 6	Feeding period 7	Feeding period 1	Feeding period 2	Feeding period 3	Feeding period 4	Feeding period 5
4	Feeding period 5	Feeding period 6	Feeding period 7	Feeding period 1	Feeding period 2	Feeding period 3	Feeding period 4
5	Feeding period 4	Feeding period 5	Feeding period 6	Feeding period 7	Feeding period 1	Feeding period 2	Feeding period 3
6	Feeding period 3	Feeding period 4	Feeding period 5	Feeding period 6	Feeding period 7	Feeding period 1	Feeding period 2
7	Feeding period 2	Feeding period 3	Feeding period 4	Feeding period 5	Feeding period 6	Feeding period 7	Feeding period 1
8	Feeding period 1	Feeding period 2	Feeding period 3	Feeding period 4	Feeding period 5	Feeding period 6	Feeding period 7
9	Feeding period 7	Feeding period 1	Feeding period 2	Feeding period 3	Feeding period 4	Feeding period 5	Feeding period 6
10	Feeding period 6	Feeding period 7	Feeding period 1	Feeding period 2	Feeding period 3	Feeding period 4	Feeding period 5

**Table 2:** Analyzed dry matter and nutrient concentrations of the basic diet.

Component	Analyzed
Dry matter (g/100 g)	93.7
	In g/100 g dry matter
Crude protein	35.0
Crude fat	9.07
Crude fiber	2.24
Crude ash	11.1
Calcium	1.89
Phosphorus	1.25
Sodium	0.69
Potassium	0.97
Magnesium	0.11
	In mg/100 g dry matter
Copper	2.13
Zinc	16.9
Iron	80.6
Manganese	4.90
Oxalate	34.6

Composition (according to the declaration of the manufacturer): Meat and animal byproducts (duck meat meal), vegetables (dried sweet potato), plant byproducts (dried tapioca, cellulose), oils and fats, flaxseed, minerals, chicory as a source of inulin (0.5%).

**Table 3:** Fatty acid composition of the basic diet, sunflower oil, fish oil, and lard used in the present study.

	Basic Diet	Basic Diet	Sunflower Oil	Fish Oil	Lard
	mg/kg Diet ^1^	% of Total Fatty Acids ^2^			
Analyzed					
Caprylic acid C 8:0	<50.0	<0.1	<0.1	<0.1	<0.1
Capric acid C 10:0	111	0.2	<0.1	<0.1	<0.1
Lauric acid C 12:0	466	0.7	<0.1	<0.1	<0.1
Myristic acid C 14:0	478	0.7	<0.1	4.5	1.5
Myristoleic acid C 14:1	78.6	0.1	<0.1	0.1	<0.1
Pentadecanoic acid C 15:0	70.6	0.1	<0.1	0.3	<0.1
Palmitic acid C 16:0	13,100	19.6	6.2	13.5	26.9
Hexadecanoic acid trans-isomers C 16:1 trans	<50.0	<0.1	<0.1	<0.1	<0.1
Palmitoleic acid C 16:1	2310	3.4	0.1	5.7	1.8
Hexadecadienoic acid C16:2 (n-4)	<50.0	<0.1	<0.1	0.7	<0.1
Hexadecatrienoic acid C16:3 (n-3)	<50.0	<0.1	<0.1	<0.1	<0.1
Margaric acid C 17:0	178	0.3	<0.1	0.4	0.3
Heptadecenoic acid C 17:1	<50.0	<0.1	<0.1	<0.1	<0.1
Stearic acid C 18:0	4440	6.6	3.6	2.6	18.2
Octadecenoic acid trans-isomers C 18:1 trans	236	0.4	<0.1	1.2	0.1
Oleic acid C 18:1	23,500	35.1	28.0	12.3	36.9
Petroselinic acid C 18:1	<50.0	<0.1	<0.1	<0.1	<0.1
cis-Vaccenic acid C 18:1	1220	1.8	0.6	5.5	2.4
Octadecadienoic acid trans-isomers C 18:2 trans	122	0.2	<0.1	0.9	<0.1
Linoleic acid C 18:2 (n-6)	15,800	23.6	59.6	0.8	9.0
Octadecatrienoic acid trans-isomers C 18:3 trans	58.8	0.1	<0.1	0.2	<0.1
alpha-Linolenic acid C 18:3 (n-3)	2900	4.3	<0.1	0.7	0.8
gamma-Linolenic acid C 18:3 (n-6)	79.8	0.1	<0.1	0.1	<0.1
Stearidonic acid C 18:4 (n-3)			<0.1	3.5	<0.1
Octadecatetraenoic acid C 18:4 (n-3)	<50.0	<0.1			
Arachidic acid C 20:0	282	0.4	0.3	<0.1	0.2
Eicosenoic acid C 20:1	311	0.5	0.2	11.2	0.8
Eicosadienoic acid C 20:2 (n-6)	98.3	0.1	<0.1	0.2	0.4
Eicosatrienoic acid C 20:3 (n-3)	<50.0	<0.1	<0.1	<0.1	<0.1
Eicosatrienoic acid C 20:3 (n-6)	94.0	0.1	<0.1	<0.1	<0.1
Arachidonic acid C 20:4 (n-6)	552	0.8	<0.1	0.3	0.2
Eicosatetraenoic acid C20:4 (n-3)	<50.0	<0.1	<0.1	0.6	<0.1
Eicosapentaenoic acid C 20:5 (n-3)	<50.0	<0.1	<0.1	13.9	<0.1
Heneicosanoic acid C 21:0	57.6	0.1	<0.1	<0.1	<0.1
Behenic acid C 22:0	174	0.3	0.8	<0.1	<0.1
Docosenoic acid trans-isomers C 22:1 trans	<50.0	<0.1	<0.1	<0.1	<0.1
Docosenoic acid C 22:1	<50.0	<0.1	<0.1	0.7	<0.1
Cetoleic acid C 22:1	<50.0	<0.1	<0.1	10.4	<0.1
Docosadienoic acid C 22:2 (n-6)	<50.0	<0.1	<0.1	<0.1	<0.1
Docosatrienoic acid C 22:3	<50.0	<0.1	<0.1	<0.1	<0.1
Docosatetraenoic acid C 22:4 (n-6)	131	0.2	<0.1	0.2	<0.1
Docosapentaenoic acid C 22:5 (n-3)	<50.0	<0.1	<0.1	0.9	<0.1
Docosapentaenoic acid C 22:5 (n-6)	<50.0	<0.1	<0.1	<0.1	<0.1
Docosahexaenoic acid C 22:6 (n-3)	<50.0	<0.1	<0.1	7.3	<0.1
Tricosanoic acid C 23:0	<50.0	<0.1	<0.1	<0.1	<0.1
Lignoceric acid C 24:0	110	0.2	0.3	<0.1	<0.1
Nervonic acid C 24:1	<50.0	<0.1	<0.1	1.1	<0.1
Calculated					
Sum saturated fatty acids	19,500	29.1	11.2	21.3	47.1
Sum monounsaturated fatty acids	27,700	41.3	28.9	48.2	42.0
Total sum fatty acids	67,000				
Sum polyunsaturated fatty acids	19,800	29.6	59.6	30.3	10.4
Sum trans fatty acids	417	0.62	<0.1	2.3	0.1
n-3 Fatty acids	2900	4.33	<0.1	26.9	0.8
n-6 Fatty acids	16,800	25.1	59.6	1.6	9.6
n-9 Fatty acids	23,800	35.5	28.2	25.3	37.7
n-6:n-3 Fatty acids ratio	5.79:1	5.79:1	>596:1	0.06:1	12:1

^1^ Quantitative measurement of the fatty acids in the total diet. ^2^ Relative amounts of the single fatty acids, expressed as % of the total fatty acids in the diet, oils, and lard. If values are reported with a “<” symbol, the analyzed values were below the respective detection limit of the laboratory.

**Table 4:** Feed and water intake as well as body weight (BW) of cats fed a low-fat basic diet without (“w/o”, control treatment) or with the addition of sunflower oil, fish oil, or lard ^1^. Means and pooled standard error of the means (SEM).

	w/o	Sunflower Oil		Fish Oil		Lard		SEM	*p*-Values		
									Sunflower Oil	Fish Oil	Lard
		0.5 g	1.0 g	0.5 g	1.0 g	0.5 g	1.0 g				
	n = 9	n = 10	n = 10	n = 10	n = 10	n = 9	n = 8				
Per Feeding Period											
Feed intake (g/day)	66.0	64.2	61.0	60.2	60.9	63.0	60.5	1.07	0.1348	0.0808	0.3153
Feed intake (g/kg BW/day)	13.8	13.3	12.5	12.3	12.6	13.0	13.0	0.30	0.1226	0.0952	0.2218
BW (kg)	4.92	4.97	4.96	4.97	4.97	4.92	4.73	0.10	0.9784	0.9885	0.4541
For the Collection Period											
Feed intake (g/day)	63.6	60.6	59.4	58.0	54.8	61.0	57.5	1.30	0.2861	0.0675	0.1115
Feed intake (g/kg BW/day)	13.2	12.6	12.2	11.9	11.4	12.6	12.3	0.35	0.2774	0.0733	0.1044
BW (kg)	4.92	4.98	4.96	4.97	4.98	4.95	4.73	0.10	0.9697	0.8897	0.2511
Water intake (g/day)	111	105	102	102	105	110	103	2.38	0.6517	0.5792	0.6542
Water intake (g/kg BW/day)	22.8	21.6	20.9	20.8	21.5	22.5	22.1	0.56	0.5129	0.5377	0.5605

^1^ The sunflower oil, fish oil, and lard were added to the basic diet at concentrations of 0.5 g/kg BW/day (“0.5 g”) and 1 g/kg BW/day (“1.0 g”).

**Table 5:** Urinary pH, urine volume, and urine composition of cats fed a low-fat basic diet without (“w/o”, control treatment) or with the addition of sunflower oil, fish oil, or lard ^1^. Means and pooled standard error of the means (SEM).

	w/o	Sunflower Oil		Fish Oil		Lard		SEM	*p-Values*		
		0.5 g	1.0 g	0.5 g	1.0 g	0.5 g	1.0 g		Sunflower Oil	Fish Oil	Lard
	n = 9	n = 10	n = 10	n = 10	n = 10	n = 9	n = 8				
pH	7.29	7.35	7.31	7.30	7.43	7.24	7.14	0.04	0.9564	0.5463	0.0915
Volume (mL/day)	30.3	28.8	28.5	27.2	25.3	30.0	28.1	1.21	0.8735	0.6059	0.9741
N (%)	4.02	4.00	3.99	3.97	4.00	3.95	4.12	0.05	0.5148	0.5604	0.3457
In µg/mL											
Calcium	54.9	56.2	49.1	46.7	47.9	50.2	60.8	2.64	0.6051	0.4229	0.3209
Phosphate	8615	8444	8465	8379	7920	8565	8905	126	0.6950	0.2159	0.2665
Sodium	6516	6548	6315	6195	6110	6010	6224	126	0.6224	0.4466	0.2934
Chloride	21,375	21,373	21,478	21,407	20,893	20,975	19,749	269	0.8770	0.6411	0.3690
Potassium	8761	9035	8728	8700	8387	8520	8903	136	0.2304	0.6332	0.3680
Magnesium	90.1	93.6	88.4	83.0	76.1	90.3	84.0	3.23	0.8291	0.1651	0.7729
Ammonium	2052	1985	2100	2133	2019	2071	2048	35.0	0.2375	0.4460	0.9871
Uric acid	108	101	121	133	119	115	121	3.50	0.0669	0.2874	0.8020
Creatinine	3766	4093	4082	4143	4134	3823	4082	86.8	0.2769	0.4050	0.0521
Sulfate	4133	4237	4056	4029	4204	4020	4293	65.3	0.5765	0.5312	0.4060
Oxalate	124	116	138	147	133	114	142	5.25	0.4864	0.2870	0.3999
Citrate	1124 ^ab^	1169	1025	914	1036	1012 ^a^	1347 ^b^	55.2	0.3610	0.1307	0.0008
Hippuric acid	1322 ^ab^	1321 ^a^	1083 ^b^	1060	1105	1122	1188	45.9	0.0403	0.1476	0.3090
RSS CaOx	2.89	3.08	3.14	3.38	2.88	2.46	3.46	0.15	0.9784	0.5207	0.1079
RSS MAP	9.25	6.06	8.42	5.57	7.33	6.76	4.62	0.98	0.5649	0.7773	*

^1^ The sunflower oil, fish oil, and lard were added to the basic diet at concentrations of 0.5 g/kg BW/day (“0.5 g”) and 1 g/kg BW/day (“1.0 g”). * As the model did not converge for this parameter and group, no *p*-value can be stated. RSS CaOx and RSS MAP: Relative supersaturation with calcium oxalate and struvite (magnesium ammonium phosphate, MAP), respectively. Different letters in the same row indicate a significant intergroup difference (*p* < 0.05; separately evaluated for the treatment groups “Sunflower oil”, “Fish oil”, and “Lard”).

**Table 6:** Renal mineral excretion of cats fed a low-fat basic diet without (“w/o”, control treatment) or with the addition of sunflower oil, fish oil, or lard ^1^. Means and pooled standard error of the means (SEM).

	w/o	Sunflower Oil		Fish Oil		Lard		SEM	*p*-Values		
		0.5 g	1.0 g	0.5 g	1.0 g	0.5 g	1.0 g		Sunflower Oil	Fish Oil	Lard
	n = 9	n = 10	n = 10	n = 10	n = 10	n = 9	n = 8				
Renal Mineral Excretion (mg/kg BW/day)											
Calcium	0.36	0.35	0.30	0.27	0.25	0.32	0.39	0.03	0.4695	0.1763	0.2364
Phosphorus	17.4	15.8	16.2	15.2	13.9	17.0	17.2	0.73	0.8492	0.4917	0.9496
Sodium	40.1	38.0	37.2	34.7	32.6	37.8	36.8	1.78	0.8700	0.2589	0.7003
Potassium	53.8	52.2	51.4	48.4	45.4	52.7	52.1	2.33	0.9660	0.5656	0.9418
Magnesium	0.58	0.57	0.52	0.47	0.41	0.56	0.54	0.03	0.6784	0.4343	0.9127
Renal Mineral Excretion/Mineral Intake (%)											
Calcium	0.16	0.16	0.13	0.13	0.13	0.14	0.17	0.01	0.5450	0.3543	0.4255
Phosphorus	11.4	11.0	11.1	10.8	10.3	11.5	11.4	0.37	0.9521	0.8807	0.9871
Sodium	46.7	47.3	45.8	44.0	42.9	45.7	43.9	1.50	0.9538	0.7307	0.9084
Potassium	45.0	46.5	45.3	44.0	42.7	45.8	44.7	1.42	0.9674	0.9365	0.9575
Magnesium	4.45	4.55	4.18	4.06	3.54	4.39	4.18	0.23	0.7705	0.6585	0.9677

^1^ The sunflower oil, fish oil, and lard were added to the basic diet at concentrations of 0.5 g/kg BW/day (“0.5 g”) and 1 g/kg BW/day (“1.0 g”).

**Table 7:** Amount of feces, fecal score, fecal dry matter, and nutrient concentrations in the feces of cats fed a low-fat basic diet without (“w/o”, control treatment) or with the addition of sunflower oil, fish oil, or lard ^1^. Means and pooled standard error of the means (SEM).

	w/o	Sunflower Oil		Fish Oil		Lard		SEM	*p*-Values		
		0.5 g	1.0 g	0.5 g	1.0 g	0.5 g	1.0 g		Sunflower Oil	Fish Oil	Lard
	n = 9	n = 10	n = 10	n = 10	n = 10	n = 9	n = 8				
Amount of feces (g/day)	33.3	27.7	32.2	28.6	29.0	33.2	30.7	1.71	0.3043	0.6370	0.8904
Amount of feces (g dry matter/day)	11.4	10.1	11.1	10.0	10.3	11.8	10.9	0.50	0.4732	0.6186	0.7994
Fecal score	2.30	2.15	2.27	2.20	2.18	2.23	2.31	0.03	0.2700	0.1209	0.2998
Dry matter (%)	35.1	37.2	35.8	37.9	37.5	36.1	37.4	0.56	0.1086	0.2005	0.3611
In mg/g Dry Matter											
Crude protein	331	327	334	330	320	336	327	2.98	0.3748	0.6075	0.4101
Crude fat	21.3 ^a^	23.8 ^ab^	26.2 ^b^	24.1 ^ab^	27.0 ^b^	23.9	27.6	0.67	0.0044	0.0029	0.0358
Crude fiber	155	151	143	148	150	160	151	2.88	0.4713	0.6984	0.8112
Crude ash	318 ^a^	315	310	309	315	307 ^ab^	305 ^b^	2.30	0.1666	0.0755	0.0050
Calcium	67.5 ^a^	70.0 ^b^	71.8 ^ab^	68.1	69.9	68.3	67.0	0.79	0.0423	0.1813	0.6804
Phosphorus	39.4 ^a^	39.2	38.2	38.2 ^b^	38.6 ^ab^	37.8 ^ab^	37.6 ^b^	0.29	0.0407	0.0213	0.0120
Sodium	1.82	1.73	1.93	1.85	1.77	1.72	1.69	0.07	0.6719	0.8863	0.7560
Potassium	2.14	2.29	2.30	2.06	2.05	1.76	2.15	0.07	0.3738	0.9198	0.0340
Magnesium	4.10	3.95	3.97	4.01	4.07	4.02	4.39	0.07	0.5611	0.6841	0.6658

^1^ The sunflower oil, fish oil, and lard were added to the basic diet at concentrations of 0.5 g/kg BW/day (“0.5 g”) and 1 g/kg BW/day (“1.0 g”). Different letters in the same row indicate a significant intergroup difference (*p* < 0.05; separately evaluated for the treatment groups “Sunflower oil”, “Fish oil”, and “Lard”).

**Table 8:** Fecal nutrient excretion and apparent nutrient digestibility of cats fed a low-fat basic diet without (“w/o”, control treatment) or with the addition of sunflower oil, fish oil, or lard ^1^. Means and pooled standard error of the means (SEM).

	w/o	Sunflower Oil		Fish Oil		Lard		SEM	*p*-Values		
		0.5 g	1.0 g	0.5 g	1.0 g	0.5 g	1.0 g		Sunflower Oil	Fish Oil	Lard
	n = 9	n = 10	n = 10	n = 10	n = 10	n = 9	n = 8				
Fecal Excretion (mg/kg BW/day)											
Crude protein	778	689	777	705	695	825	784	43.1	0.3889	0.6643	0.4993
Crude fat	49.7	47.8	60.9	50.8	57.1	59.2	63.3	3.39	0.2531	0.5100	0.2984
Crude fiber	361	318	318	304	316	390	341	17.4	0.4195	0.4023	0.5747
Crude ash	738	650	700	624	653	746	717	32.8	0.5317	0.4376	0.1013
Calcium	157	144	170	139	145	167	157	8.35	0.4762	0.6641	0.0387
Phosphorus	91.6	80.7	86.3	76.8	80.7	92.1	88.1	4.04	0.5682	0.4283	0.1634
Sodium	4.17	3.38	4.62	4.16	3.74	4.21	4.18	0.30	0.2928	0.8901	0.8622
Potassium	5.12	4.91	5.22	4.15	4.68	4.54	5.35	0.32	0.8423	0.2613	0.4359
Magnesium	9.61	8.12	8.93	8.24	8.47	9.79	9.92	0.44	0.3083	0.4543	0.8624
Apparent Digestibility (%)											
Crude protein	82.0	83.6	81.2	82.7	81.7	80.3	81.5	0.72	0.4593	0.9384	0.5824
Crude fat	95.6	95.5	94.3	95.1	94.0	94.6	94.1	0.24	0.2294	0.1762	0.1309
Crude ash	45.5	50.9	46.0	51.2	44.3	43.8	46.0	1.76	0.6047	0.6537	0.3388
Calcium	32.2	35.7	24.9	36.7	28.0	26.4	30.5	2.51	0.5690	0.6106	0.1414
Phosphorus	39.7	45.8	40.8	46.5	39.1	38.4	40.8	1.94	0.6531	0.6401	0.4076
Sodium	94.9	95.9	94.3	94.9	94.9	94.9	95.1	0.31	0.3343	0.9993	0.7766
Potassium	95.9	95.8	95.4	96.3	95.7	96.3	95.5	0.20	0.5067	0.5206	0.4554
Magnesium	27.3	35.9	28.2	33.3	25.2	23.1	21.5	2.49	0.5019	0.7500	0.7731

^1^ The sunflower oil, fish oil, and lard were added to the basic diet at concentrations of 0.5 g/kg BW/day (“0.5 g”) and 1 g/kg BW/day (“1.0 g”).

**Table 9:** Clinical–chemical parameters in the plasma of cats fed a low-fat basic diet without (“w/o”, control treatment) or with the addition of sunflower oil, fish oil, or lard ^1^. Means and pooled standard error of the means (SEM).

	w/o	Sunflower Oil		Fish Oil		Lard		SEM	*p*-Values		
		0.5 g	1.0 g	0.5 g	1.0 g	0.5 g	1.0 g		Sunflower Oil	Fish Oil	Lard
	n = 9	n = 10	n = 10	n = 10	n = 10	n = 9	n = 8				
AP (U/L)	33.1	37.3	36.4	32.6	33.8	34.7	34.5	1.31	0.1858	0.7910	0.1231
LDH (U/L)	119	90.0	83.3	115	108	101	98.9	4.51	0.0840	0.8658	0.6659
Cholesterol (mmol/L)	3.24 ^a^	3.30	3.20	3.41 ^ab^	3.70 ^b^	3.21	3.48	0.09	0.2071	0.0186	0.6598
HDL cholesterol (mg/dL)	101	104	102	101	105	102	108	2.42	0.2918	0.3126	0.6719
LDL cholesterol (mg/dL)	19.4 ^ab^	21.1	20.7	22.6 ^a^	26.8 ^b^	19.4	22.5	1.39	0.4075	0.0167	0.8873
Triglycerides (mmol/L)	0.44 ^a^	0.44	0.44	0.39 ^b^	0.41 ^ab^	0.43	0.44	0.01	0.9831	0.0268	0.6622
NEFAs (mmol/L)	0.43	0.50	0.46	0.43	0.41	0.48	0.44	0.01	0.3023	0.5799	0.0008
Total protein (g/L)	73.2 ^ab^	73.4	73.3	74.0 ^a^	71.8 ^b^	73.2	71.8	0.53	0.9161	0.0177	0.1281
Urea (mmol/L)	8.91	8.59	8.58	8.34	8.46	8.57	8.53	0.10	0.1230	0.1088	0.1197
Creatinine (µmol/L)	135	136	138	137	142	135	139	2.26	0.7485	0.4512	0.0375
Calcium (mmol/L)	2.61	2.62	2.63	2.60	2.63	2.57	2.60	0.02	0.8633	0.7588	0.5799
Phosphate (mmol/L)	1.28 ^a^	1.29	1.34	1.41 ^ab^	1.47 ^b^	1.31	1.24	0.02	0.4860	0.0304	0.3565

^1^ The sunflower oil, fish oil, and lard were added to the basic diet at concentrations of 0.5 g/kg BW/day (“0.5 g”) and 1 g/kg BW/day (“1.0 g”). AP: alkaline phosphatase; LDH: lactate dehydrogenase; HDL: high-density lipoprotein; LDL: low-density lipoprotein; NEFAs: non-esterified fatty acids. Different letters in the same row indicate a significant intergroup difference (*p* < 0.05; separately evaluated for the treatment groups “Sunflower oil”, “Fish oil”, and “Lard”). Reference values (Laboklin, Bad Kissingen, Germany): AP: <65 U/L, LDH: <108 U/L, cholesterol: 1.8–3.9 mmol/L, triglycerides: <1.14 mmol/L, total protein: 57–94 g/L, urea: 5.0–11.3 mmol/L, creatinine: <168 µmol/L, calcium: 2.3–3.0 mmol/L, phosphate: 0.8–1.9 mmol/L.

## Data Availability

The raw data supporting the conclusions of this article will be made available by the authors upon request.
